# Effect of breathing intervention in patients with COVID and healthcare workers

**DOI:** 10.3389/fpubh.2022.945988

**Published:** 2022-09-30

**Authors:** Manjari Rain, Goverdhan Dutt Puri, Aashish Bhalla, Pramod Avti, Balachundhar Subramaniam, Vipin Kaushal, Vinod Srivastava, Pranay Mahajan, Mini Singh, Navin Pandey, Pankaj Malhotra, Sonu Goel, Krishan Kumar, Naresh Sachdeva, Kalyan Maity, Prashant Verma, Nishant Dixit, Sheetal Jindal Gupta, Priya Mehra, Pooja Nadholta, Radhika Khosla, Shweta Ahuja, Akshay Anand

**Affiliations:** ^1^Department of Neurology, Post Graduate Institute of Medical Education and Research, Chandigarh, India; ^2^Department of Anesthesia, Post Graduate Institute of Medical Education and Research, Chandigarh, India; ^3^Department of Internal Medicine, Post Graduate Institute of Medical Education and Research, Chandigarh, India; ^4^Department of Biophysics, Post Graduate Institute of Medical Education and Research, Chandigarh, India; ^5^Harvard Medical School, Beth Israel Deaconess Medical Center, Boston, MA, United States; ^6^Department of Hospital Administration, Post Graduate Institute of Medical Education and Research, Chandigarh, India; ^7^College of Health and Behavioral Sciences, Fort Hays State University, Hays, KS, United States; ^8^Department of Virology, Post Graduate Institute of Medical Education and Research, Chandigarh, India; ^9^Department of Community Medicine and School of Public Health, Post Graduate Institute of Medical Education and Research, Chandigarh, India; ^10^Department of Psychiatry, Post Graduate 30 Institute of Medical Education and Research, Chandigarh, India; ^11^Department of Endocrinology, Post Graduate Institute of Medical Education and Research, Chandigarh, India; ^12^Swami Vivekananda Yoga Anusandhana Samsthana, Bengaluru, India; ^13^Interdisciplinary Centre for Swami Vivekananda Studies, Panjab University, Chandigarh, India; ^14^Department of Psychology, Panjab University, Chandigarh, India; ^15^Department of Biotechnology, Panjab University, Chandigarh, India; ^16^Spectra Health Care, Chandigarh, India; ^17^Centre of Phenomenology and Cognitive Sciences, Panjab University, Chandigarh, India; ^18^CCRYN-Collaborative Center for Mind Body Intervention Through Yoga, Post Graduate Institute of Medical Education and Research, Chandigarh, India

**Keywords:** COVID-19, breathing technique, D-dimer, mindfulness, yoga, SpO_2_

## Abstract

**Background:**

Regulated breathing facilitates ventilation and reduces breathlessness. However, the effect of Yogic breathing on patients with COVID remains unclear. We aimed to evaluate the efficacy of two breathing protocols, i.e., short breathing technique (SBT) and long duration breathing technique (LBDT).

**Methods:**

Three groups including COVID-positive patients, COVID-recovered patients, and healthcare workers (HCWs) were included in the study and segregated into Yoga and control groups. SBT was administered to COVID-positive patients. Both SBT and LBDT were administered to COVID-recovered patients and HCWs. A total of 18 biochemical parameters, a 6-min walk test (6MWT), and a 1-min sit-stand test (1MSST) were assessed on 0th, 7th, and 15th days, where biochemical parameters were the primary outcome. Pre-post estimation of neuropsychological parameters (nine questionnaires) and heart rate variability (HRV) were carried out. The paired *t*-test or Wilcoxon rank test was applied for pre-post comparison and the Student's *t*-test or Mann–Whitney *U* test was used for group comparison. Repeated measures test was applied for data recorded at three time points.

**Results:**

A significant elevation in white blood cell (WBC) count was observed in COVID-positive intervention (*p* < 0.001) and control groups (*p* = 0.003), indicating no role of intervention on change in WBC number. WBC count (*p* = 0.002) and D-dimer (*p* = 0.002) significantly decreased in the COVID-recovered intervention group. D-dimer was also reduced in HCWs practicing Yogic breathing as compared to controls (*p* = 0.01). D-dimer was the primary outcome, which remained below 0.50 μg/ml (a cutoff value to define severity) in the COVID-positive yoga group (CYG) and decreased in the COVID-recovered yoga group (RYG) and the HCW yoga group (HYG) after intervention. A 6-min walk test (6MWT) showed an increase in distance covered among the COVID-positive patients (*p* = 0.01) and HCWs (*p* = 0.002) after intervention. The high-frequency power (*p* = 0.01) was found to be reduced in the COVID-positive intervention group. No significant change in neuropsychological parameters was observed.

**Conclusion:**

Yogic breathing lowered D-dimer, which is helpful in reducing thrombosis and venous thromboembolism in patients with COVID-19 besides lowering the chances of vaccine-induced thrombotic thrombocytopenia in vaccinated individuals. The breathing intervention improved exercise capacity in mild to moderate cases of COVID-19. Further studies can show if such breathing techniques can influence immunity-related genes, as reported recently in a study. We suggest that Yogic breathing may be considered an integrative approach for the management of patients with COVID.

**Trial registration:**

http://ctri.nic.in/Clinicaltrials/login.php, identifier: CTRI/2020/10/028195.

## Introduction

Prevention of COVID-19 and its management has been a daunting task worldwide. Post-COVID complications have also posed several challenges with children being most vulnerable to COVID infections (1). The ongoing public health crisis associated with new variants of COVID-19 and comorbidities (heart disease, diabetes, respiratory problems, and immune disorders) has escalated the fatality rates, adversely impacting the resource-deficient countries with the large population (2–4). The possibility of COVID-like outbreaks appears more likely than previously thought, and it is likely that previously developed vaccines will not be as effective for new variants like omicron (5, 6).

An integrative treatment protocol combining traditional knowledge with modern medicine is being developed in India to manage COVID-19 (7, 8). In some recent studies, various treatment modalities have been proposed to test the effectiveness of yoga and meditation; however, the outcome measures have remained limited to subjective measures (2, 5, 7, 9).

Popular practices like yoga, meditation, and Ayurveda are often argued as affordable adjunct therapies that may reduce the risks of infectious diseases associated with respiratory distress (2, 5, 7, 9–13). A deep breathing technique has also been shown to have promising results in COVID-19 management with a few physicians using it for patients with COVID-19 (14). Yogic breathing techniques are believed to facilitate ventilation and are instrumental in reducing breathlessness, thereby bringing comfort to patients in isolation (15–17). Such techniques alleviate stress, anxiety, and psychosomatic symptoms, improve cognition, and are useful in the management of diabetes and hypertension (18–24). It has been previously shown that meditation and yoga result in a decrease in hemoglobin A1c (HbA1C, a diabetes biomarker) levels, inflammation, and oxidative stress besides improving the lipid profile in diabetes and hypertension, contributing to COVID-19 risk reduction (20–23). These comorbid conditions are well known to exacerbate the severity of COVID-19 (25–27). Therefore, breathing techniques that can improve the respiratory capacity are ideal for studying their efficacy in COVID-19 management.

This study analyzes the effectiveness of yogic breathing techniques, i.e., short breathing technique (SBT) and longer duration breathing technique (LDBT), in the management of COVID-19. We aimed to investigate the effect of 15-day yogic breathing intervention (short and long duration) during COVID-19 infection, post-COVID-19 infection, and among healthcare workers (HCWs), all of whom are vulnerable to the disease while working in COVID-19 wards. The results were obtained by analyzing neuropsychological and biochemical parameters in tandem with the assessment of heart rate variability (HRV) and 6-min walk test (6MWT). A 1-min sit-stand test (1MSST) that is a reliable triage marker was also included in this study (28). A control non-intervention group was also included. In addition, to test the interindividual variability in disease presentation and different outcomes of intervention, we clinically stratified the study participants into different *Prakriti*, which is a basic constitution of an individual described in Ayurveda, an ancient Indian medical system. *Prakriti* of an individual renders a person to certain personality and physiological outcomes, thus predisposing them to certain health conditions (16, 17). Recent studies have reported genetic predisposition for COVID-19 infection, progression, and immune response (29–34). It is to be noted here that human leukocyte antigen (HLA), which is associated with COVID-19, has varying genotype across different *Prakriti* types (33–35). Interestingly, biochemical profiles, such as biomarkers of liver function, lipid profiles, and hemoglobin (Hb), which are readily checked in patients with COVID-19, differ among *Prakriti* types (36). Thus, evaluating clinical and biochemical parameters based on *Prakriti* can provide important insights into the treatment of COVID-19.

*Prakriti* has been described to be the result of the interaction of genetic and environmental factors and has a difference in molecular framework involving transport, regulation of cyclin-dependent protein kinase activity, immune response, and regulation of blood coagulation as reported by a whole-genome expression study (36, 37). *Prakriti* is widely believed to be inheritable (38). Individuals were assigned to different *Prakriti* types or *doshas*, namely, the *Kapha, Pitta, Vata*, and combination of these three by a qualified practitioner of Ayurveda based on their clinical phenotypes such as hair color, skin type, behavioral, and lifestyle preferences. Each of this dosha is responsible for a specific physiological process. Broadly, *Kapha* regulates lubrication, cohesion, and structure; *Pitta* regulates energy; and *Vata* regulates movements (39).

The biochemical parameters were the primary outcome of the study as some of these were routinely tested among patients with COVID-19 to define severity and modify treatment accordingly.

## Methods

### Study subjects

The study was registered under the Clinical Trials Registry – India (CTRI/2020/10/028195) on 1 October 2020. The study subjects were recruited from Nehru Hospital Extension (NHE), Postgraduate Institute of Medical Education and Research (PGIMER), Chandigarh, India, after the approval from the Institutes Ethics Committee (IEC no. IEC-05/2020-1646), between October 2020 and January 2021. Three groups were identified for this study, namely, (1) COVID-positive group included patients who were confirmed positive for severe acute respiratory syndrome coronavirus 2 (SARS-CoV-2) at the time of the recruitment, (2) COVID-recovered group represented those who were positive at least 1 month before the recruitment, and (3) HCW group comprised those individuals working at NHE/COVID-19 block of PGIMER, Chandigarh, India. HCWs were negative for COVID-19 before and during the study. The COVID-positive group was followed for 45 more days to document any episode of recurrence. The details of admitted COVID-positive patients were provided by NHE staff daily; the patients were approached in person to explain the study and recruited after consent was obtained. The recruitment in the COVID-positive group was carried out following the Institute's Ethical Guidelines under PPE protection. NHE staff also provided the details of previously admitted COVID patients (who had recovered); these patients were contacted over the phone and recruited under the COVID-recovered group after consent was obtained. HCWs working at NHE were recruited under the third group after obtaining consent.

Furthermore, each group was randomly divided into two subgroups by manual chit-picking method; the subgroups included an intervention or yoga group and a non-intervention or control group. The groups were COVID-positive control group (CCG), COVID-positive yoga group (CYG), COVID-recovered control group (RCG), COVID-recovered yoga group (RYG), HCW control group (HCG), and HCW yoga group (HYG). The participants' age varied from 20 to 65 years. The subjects were not recruited if they were yoga practitioners or refused to give consent. Patients with COVID-19 on ventilators, not cleared by treating clinicians for the breathing yoga protocols, or with epilepsy, brain tumor, brain aneurysm, pregnancy, and other critical health conditions were excluded.

### Intervention: Breathing techniques

The protocol for breathing intervention was based on the theoretical framework of Yogic scriptures and is harmless. It included two techniques, namely, SBT (Supplementary Table 1, Supplementary Video 1) and LDBT (Supplementary Video 2) (40). SBT was administered in the morning to the COVID-19 yoga group for 15 days. SBT and LDBT was administered in the morning and evening, respectively, to the yoga group of COVID-recovered and HCWs for 15 days. Certified yoga trainers administered yogic breathing intervention *via* video to subjects with COVID-positive and *via* video calls to other two groups. Videos and/or pictures of the interventions were recorded, and records were retained following the approved research protocols by researchers to increase the data credibility and validity.

SBT, LDBT, or any other forms of exercise were not provided to the control group. Controls were under usual clinical care and were not wait-listed for intervention.

### Data collection and quality control

Videos and/or pictures were captured during randomization and data collection for transparency and fidelity measurement. The data were compiled on Microsoft Excel, and the entries were validated twice by two blinded researchers, before analysis, in order to maintain the accuracy of curated data. The validated data were subjected to various quality control measures based on the data type. Hence, the sample size was shown differently for each parameter.

Many participants did not give consent to the 7th-day blood sampling, which resulted in performing the analysis for the baseline, and the 15th day for the biomarker analysis. One participant in HYG was excluded from biomarker analysis as 15th-day sampling could not be done due to drop out of the participant.

Neuropsychological data were quality controlled by excluding the participants who did not attempt to complete ≥80% of the total pre- or post-questionnaires. Each questionnaire with over 80% answered questions was considered for analysis. Post-Traumatic Growth Inventory (PTGI) was not considered for estimating the cutoff value because all participants did not experience trauma or considered COVID-19 to be a traumatic situation.

For 6MWT and 1MSST, participants were considered for repeated measure analysis when measurements were made on all three time points. The sample size was the same for 6MWT and 1MSST, except for one participant of RYG, who could not perform 1MSST on the 15th day due to health issues. Hence, he was excluded from the 1MSST analysis. Similarly, HRV data were considered if the recording of 360 s was available for baseline and 15^th^ day. The final sample size is mentioned in each Table and Figure.

### Biomarker estimation

The included blood markers were routinely checked for monitoring the health of patients. Hence, these biochemical parameters were the primary outcome of the study and the remaining parameters were the secondary outcome. Several biomarker tests, namely, complete blood count (CBC), Hb, activated partial thromboplastin time (aPTT), D-dimer, cortisol, procalcitonin, liver function test (LFT), and renal function test (RFT) were conducted on the baseline, 7th, and 15th days for control and yoga groups. LFT included alanine transaminase (ALT), aspartate transaminase (AST), conjugated bilirubin, total bilirubin, and alkaline phosphatase (ALP) tests, and RFT included creatinine, C-reactive protein (CRP), calcium, and urea.

An automated cell counter was used for CBC, Hb, and aPTT. D-dimer was estimated by Nephelometry using a MISPA-i2 detection kit (AGAPPE Diagnostic Ltd., India). Elecsys BRAHMS PCT and Elecsys CORTISOL kit (Roche Molecular Diagnostics, USA) were used to detect procalcitonin and cortisol, respectively. The enzymatic assay was done for detecting ALT, AST, and ALP (Arkray, Japan). In addition, bilirubin (conjugated and total) was detected by the Diazo method using a T&D Bilirubin kit (Arkray, Japan). The urease method was used for detecting urea (Arkray, Japan) and the Jaffe's method was used for estimating creatinine (Q-line, POCT Services Pvt Ltd., India). The o-cresolphthalein complexone (OCPC) method was used for estimating calcium (Arkray, Japan), and Turbigold CRP Test Kit (Arkray, Japan) was used for estimating CRP.

### Neuropsychological and *Prakriti* assessments

Mental wellbeing, wellness, resilience, and quality of life were measured using questionnaires at the baseline and on the 15th day. Stress was measured by the Perceived Stress Score (PSS), and changes in mood states were measured by Profile of Mood States (POMS). The Mindful Attention Awareness Scale (MAAS) was used to evaluate mindfulness, and the Dispositional Positive Emotion Scales – JOY subscale (DPES-JOY) was administered to assess joy. Anxiety and depression were assessed by the Patient Health Questionnaires (PHQ-4), and the Warwick-Edinburgh Wellbeing Scale (WEMWBS) was used to evaluate patients' overall wellbeing. The Brief Resilience Scale (BRS) and PTGI measured resilience (toward any trauma). Quality of life was estimated by using the WHO Quality of Life (WHOQOL) scale. WHOQOL has four domains, where Domain one represents physical health, Domain two measures psychological health, Domain three corresponds to social relationships, and Domain four assesses environmental health. *Prakriti* was assessed by the Sushrutha Prakriti Inventory Questionnaire.

### Six-min walk test (6MWT)

A 6MWT was performed to assess the aerobic exercise capacity and endurance on baseline, 7th, and 15th days as per the published protocol (41). The specific distance was measured using a measuring tape, and two ends were marked on the available space. All participants were requested to walk for 6 min between the two points without talking or using the phone. Cardiovascular physiological parameters like pulse rate (PR), oxygen saturation (SpO_2_) (Crosso Pulse Oximeter, India), and blood pressure (Morepen Laboratories Ltd., India) were monitored before and after the walk. The covered distance was recorded for each participant, and the participants were requested to rate how much tiredness/breathlessness they felt during the walk using the Borg Breathless Scale, ranging between 0 and 10.

### One-min sit and stand test (1MSST)

The participants performed the 1MSST on a standard chair/stool with a height of 46–48 cm. The chair was without an armrest and had a flat seat. Participants sat upright straight with their knees bent to 90° on the chair with folded hands in front to avoid taking any support during movement (42). A stopwatch was used to measure participants' cycles of one sit and one stand for 1 min. Participants were instructed to stand straight and sit straight on the chair with their bottom touching the seat without leaning back. SpO_2_, PR, and blood pressure were recorded before and after the test on the baseline, 7th, and 15th days.

### Heart rate variability (HRV)

Electrocardiogram (ECG) captured on the baseline and 15th day was used to analyze HRV. MP45 HRV Machine (BIOPAC systems, Inc., CA, USA) that used the Biopac Student Lab software version 4.1.1 and Physiograph-D (Recorders Medicare Systems Pvt. Ltd., India) that used the Physiograph-D software were used to calculate HRV. The MP45 HRV instrument was used in the COVID-positive group.

### Statistical analysis

IBM SPSS version 21.0 was used for statistical analysis. A paired *t*-test or Wilcoxon rank test was applied to estimate significance in blood markers and neuropsychological parameters, depending upon whether the data are normal or non-normal. Similarly, subgroup comparisons were performed by the Student's *t*-test or Mann–Whitney *U*-test. A repeated measure was used to evaluate the change in the difference between before-test and after-test measures of SpO_2_, PR, and blood pressure for 1MSST and 6MWT. In 6MWT, the difference in distance was evaluated by ANOVA and Tukey's *post-hoc* test. The significance level was *p*-value ≤ 0.05. P-values were adjusted with age and gender for comparisons between the subgroups and correlations. The Bonferroni correction for multiple comparisons was performed wherever applicable.

## Results

### Study population and setting

**Figure 1:**
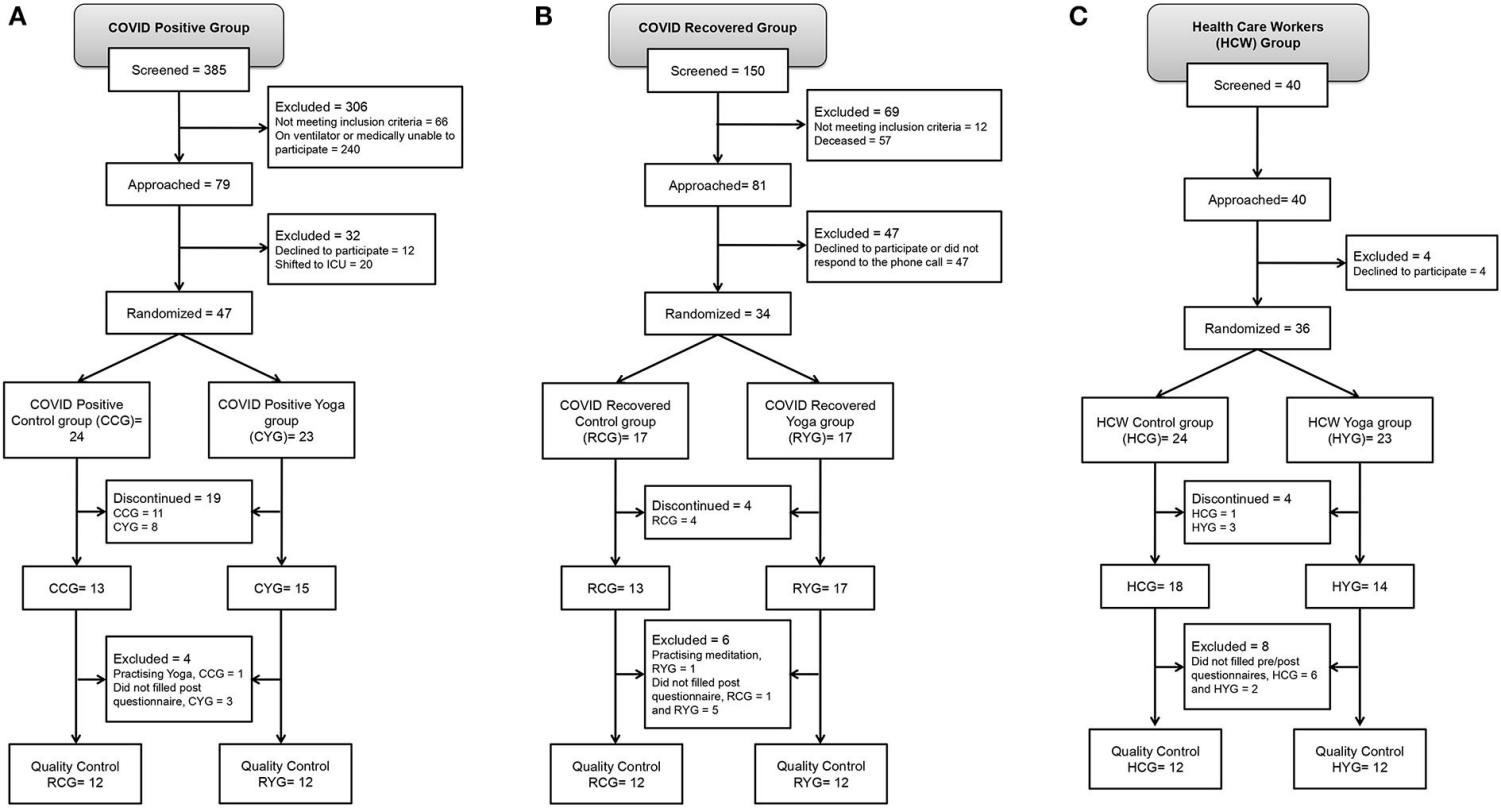
Study participant recruitment as per CONSORT. **(A)** COVID-positive group, **(B)** COVID-recovered group and **(C)** HCW group.

The data for the patients with COVID-19 were acquired from NHE, PGIMER, Chandigarh, India, from which COVID-positive and COVID-recovered subjects were recruited. HCWs posted at NHE were also recruited in the third group. The details of enrolment and 6 subgroups are summarized in Figure 1. The sociodemographic data included age, body mass index (BMI), gender, smoking and alcohol consumption, diet, physical activity, and duration of sleep. A significant difference was observed in the age of RCG and RYG (*p* = 0.04) and the diet of HCG and HYG (*p* = 0.03) (Supplementary Table 2). None of the COVID-positive patients showed recurrence of COVID-19 after 45 days of study.

### Effect of intervention on blood parameters

A significant increase in WBC count was observed in CYG and CCG, as the condition of patients improved in both the subgroups (Table 1). WBC count significantly decreased in RYG after intervention (Table 1). D-dimer was found high in RYG at baseline, which was reduced after the intervention (Table 1). In the HCW group, ALP was found to be decreased significantly after the intervention, while ALT was found to be increased, and creatinine was found to be decreased significantly in the control subgroup (Table 1). The low levels of cortisol were observed in HYG than in HCG on the 15th day (Table 1). The cutoff value for significance was 0.003 after the Bonferroni correction.

**Table 1:** Biochemical parameters in the three study groups.

**Biomarkers**	**Study groups**	**Baseline** **Mean (SD)**	**15th day Mean (SD)**	**Baseline vs. 15th day** **(*P*-value)**	**Yoga vs. controls ^a^At baseline** **^b^ On 15th day (*P*-value)**	**Yoga vs. controls** **^a^At baseline** **^b^ On 15th day** **(Adjusted *P*-value)**
White blood cell (WBC) count, (x10^9^ /L)	CCG	5.10 (1.75)	7.24 (2.23)	0.003	^a^ 0.53 ^b^0.46	^a^ 0.72 ^b^ 0.50
	CYG	4.69 (1.07)	6.58 (1.72)	< 0.001		
	RCG	7.26 (1.77)	6.48 (1.32)	0.06	^a^0.83 ^b^0.93	^a^ 0.85 ^b^ 0.86
	RYG	7.11 (1.64)	6.53 (1.31)	0.002		
	HCG	8.33 (2.01)	8.48 (2.64)	0.78	^a^ 0.04 ^b^ 0.06	^a^ 0.06 ^b^ 0.14
	HYG	6.49 (2.03)	6.57 (1.86)	0.85		
Red blood cell (RBC) count, (x10^12^/L)	CCG	4.64 (1.03)	4.49 (0.96)	0.33	^a^ 0.50 ^b^ 0.63	^a^ 0.48 ^b^ 0.79
	CYG	4.88 (0.39)	4.66 (0.60)	0.20		
	RCG	4.55 (0.72)	4.57 (0.65)	0.58	^a^ 0.97 ^b^ 0.89	^a^ 0.93 ^b^ 0.76
	RYG	4.54 (0.48)	4.60 (0.49)	0.35		
	HCG	4.41 (0.35)	4.34 (0.34)	0.08	^a^ 0.20 ^b^ 0.15	^a^ 0.69 ^b^ 0.55
	HYG	4.65 (0.53)	4.59 (0.46)	0.15		
Hemoglobin, (gm/dL)	CCG	13.37 (2.61)	13.20 (2.48)	0.53	^a^ 0.58 ^b^ 0.63	^a^ 0.52 ^b^ 0.53
	CYG	13.91 (1.79)	13.66 (1.89)	0.33		
	RCG	13.18 (1.85)	13.17 (1.71)	0.94	^a^ 0.70 ^b^ 0.83	^a^ 0.73 ^b^ 0.85
	RYG	12.91 (1.50)	13.03 (1.50)	0.46		
	HCG	12.68 (2.11)	12.36 (2.03)	0.02	^a^ 0.31 ^b^ 0.23	^a^ 0.87 ^b^ 0.87
	HYG	13.53 (1.77)	13.36 (1.80)	0.12		
Hematocrit (HCT)/packed cell volume (PCV), (%)	CCG	40.83 (7.98)	38.65 (6.99)	0.16	^a^ 0.60 ^b^ 0.69	^a^ 0.53 ^b^ 0.90
	CYG	42.35 (4.71)	39.73 (5.14)	0.14		
	RCG	37.65 (5.65)	34.69 (10.73)	0.39	^a^ 0.69 ^b^ 0.52	^a^ 0.81 ^b^ 0.68
	RYG	36.83 (4.11)	36.83 (4.02)	1.00		
	HCG	36.41 (5.42)	35.61 (5.36)	0.01	^a^ 0.18 ^b^ 0.19	^a^ 0.77 ^b^ 0.78
	HYG	39.50 (5.33)	38.56 (4.96)	0.04		
Platelet (thrombocyte) count, (lakh/mm^3^)	CCG	2.31 (0.93)	2.62 (0.46)	0.33	^a^ 0.77 ^b^ 0.87	^a^ 0.69 ^b^ 0.77
	CYG	2.21 (0.43)	2.68 (0.84)	0.10		
	RCG	2.44 (0.63)	2.27 (0.45)	0.19	^a^ 0.12 ^b^ 0.46	^a^ 0.35 ^b^ 0.83
	RYG	2.08 (0.44)	2.13 (0.47)	0.54		
	HCG	2.28 (0.95)	2.18 (0.89)	0.16	^a^ 0.94 ^b^ 0.89	^a^ 0.86 ^b^ 0.96
	HYG	2.25 (0.52)	2.13 (0.41)	0.21		
D-dimer, μg/ml	CCG	0.26 (0.19)	0.50 (0.58)	0.23	^a^ 0.61 ^b^ 0.47	^a^ 0.76 ^b^ 0.71
	CYG	0.22 (0.07)	0.30 (0.26)	0.46		
	RCG	0.31 (0.10)	0.25 (0.15)	0.25	^a^ 0.01 ^b^ 0.45	^a^ 0.02 ^b^ 0.19
	RYG	0.54 (0.23)	0.21 (0.12)	0.002		
	HCG	0.36 (0.25)	0.39 (0.22)	0.65	^a^ 0.77 ^b^ 0.01	^a^ 0.55 ^b^ 0.01
	HYG	0.42 (0.67)	0.12 (0.21)	0.17		
Activated partial thromboplastin (aPTT), time (sec)	CCG	30.86 (3.09)	27.55 (2.63)	0.01	^a^ 0.52 ^b^ 0.65	^a^ 0.58 ^b^ 0.90
	CYG	32.33 (4.92)	28.65 (5.69)	0.04		
	RCG	26.05 (2.78)	25.35 (1.92)	0.40	^a^ 0.99 ^b^ 0.98	^a^ 0.90 ^b^ 0.92
	RYG	26.07 (2.19)	25.32 (2.29)	0.30		
	HCG	23.26 (1.63)	24.43 (1.14)	0.13	^a^ 0.57 ^b^ 0.82	^a^ 0.21 ^b^ 0.72
	HYG	23.83 (2.88)	24.58 (1.83)	0.50		
Total Bilirubin, mg/dL	CCG	0.55 (0.30)	0.77 (0.53)	0.06	^a^ 0.76 ^b^ 0.33	^a^ 0.66 ^b^ 0.32
	CYG	0.59 (0.42)	0.60 (0.18)	0.91		
	RCG	0.72 (0.20)	0.71 (0.22)	0.88	^a^ 0.89 ^b^ 0.63	^a^ 0.64 ^b^ 0.90
	RYG	0.74 (0.30)	0.77 (0.37)	0.66		
	HCG	0.64 (0.25)	0.73 (0.30)	0.07	^a^ 0.22 ^b^ 0.36	^a^ 0.51 ^b^ 0.64
	HYG	0.80 (0.37)	0.89 (0.53)	0.28		
Conjugated Bilirubin, mg/dL	CCG	0.23 (0.07)	0.25 (0.12)	0.32	^a^ 0.96 ^b^ 0.53	^a^ 0.55 ^b^ 0.43
	CYG	0.23 (0.09)	0.23 (0.08)	0.94		
	RCG	0.24 (0.07)	0.26 (0.08)	0.48	^a^ 0.34 ^b^ 0.27	^a^ 0.28 ^b^ 0.44
	RYG	0.27 (0.07)	0.32 (0.15)	0.16		
	HCG	0.22 (0.12)	0.28 (0.14)	0.02	^a^ 0.13 ^b^ 0.75	^a^ 0.28 ^b^ 0.51
	HYG	0.34 (0.22)	0.27 (0.09)	0.17		
C-reactive protein (CRP), mg/l	CCG	8.53 (8.12)	1.88 (2.37)	0.03	^a^ 0.06 ^b^ 0.85	^a^ 0.15 ^b^ 0.83
	CYG	2.84 (3.06)	2.06 (1.71)	0.42		
	RCG	4.01 (4.94)	2.52 (3.12)	0.12	^a^ 0.19 ^b^ 0.27	^a^ 0.16 ^b^ 0.19
	RYG	1.96 (1.56)	1.41 (1.47)	0.14		
	HCG	3.42 (4.34)	3.42 (4.63)	1.00	^a^ 0.15 ^b^ 0.18	^a^ 0.32 ^b^ 0.41
	HYG	1.36 (1.56)	1.37 (1.73)	0.96		
Alkaline phosphatase (ALP), U/L	CCG	76.64 (25.65)	123.45 (38.54)	0.01	^a^ 0.65 ^b^ 0.42	^a^ 0.30 ^b^ 0.92
	CYG	81.50 (24.56)	107.08 (54.13)	0.04		
	RCG	145.83 (38.46)	156.42 (35.63)	0.07	^a^ 0.52 ^b^ 0.70	^a^ 0.80 ^b^ 0.95
	RYG	156.50 (41.23)	162.75 (42.40)	0.48		
	HCG	192.83 (51.30)	171.08 (52.85)	0.03	^a^ 0.12 ^b^ 0.26	^a^ 0.03 ^b^ 0.19
	HYG	163.00 (33.00)	150.18 (28.60)	0.001		
Alanine transaminase (ALT), U/L	CCG	28.89 (21.18)	29.25 (8.68)	0.95	^a^ 0.91 ^b^ 0.10	^a^ 0.38 ^b^ 0.11
	CYG	28.05 (13.41)	44.71 (29.20)	0.08		
	RCG	22.17 (8.58)	23.67 (11.84)	0.62	^a^ 0.10 ^b^ 0.49	^a^ 0.26 ^b^ 0.94
	RYG	35.92 (25.13)	28.00 (17.92)	0.32		
	HCG	26.00 (12.08)	37.67 (10.56)	0.001	^a^ 0.57 ^b^ 0.27	^a^ 0.87 ^b^ 0.55
	HYG	29.27 (15.30)	49.00 (31.06)	0.004		
Aspartate transaminase (AST), U/L	CCG	34.82 (37.88)	23.79 (6.58)	0.28	^a^ 0.39 ^b^ 0.16	^a^ 0.85 ^b^ 0.12
	CYG	25.13 (6.06)	29.52 (11.79)	0.30		
	RCG	21.42 (3.85)	22.33 (10.98)	0.76	^a^ 0.41 ^b^ 0.59	^a^ 0.88 ^b^ 1.00
	RYG	23.83 (9.29)	24.25 (5.14)	0.85		
	HCG	24.75 (8.50)	34.75 (21.97)	0.09	^a^ 0.81 ^b^ 0.46	^a^ 0.31 ^b^ 0.35
	HYG	24.00 (6.07)	29.55 (7.41)	0.01		
Creatinine, mg/dL	CCG	1.29 (1.70)	1.00 (0.49)	0.56	^a^ 0.07 ^b^ 0.13	^a^ 0.33 ^b^ 0.26
	CYG	0.71 (0.13)	0.75 (0.23)	0.46		
	RCG	0.96 (0.13)	0.92 (0.15)	0.36	^a^ 0.92 ^b^ 0.60	^a^ 0.82 ^b^ 0.26
	RYG	0.95 (0.15)	0.88 (0.19)	0.03		
	HCG	0.95 (0.17)	0.84 (0.12)	0.002	^a^ 0.39 ^b^ 0.19	^a^ 0.84 ^b^ 0.60
	HYG	1.01 (0.16)	0.93 (0.19)	0.01		
Urea, mg/dL	CCG	34.48 (35.98)	31.73 (23.74)	0.81	^a^ 0.04 ^b^ 0.46	^a^ 0.27 ^b^ 0.68
	CYG	21.24 (2.53)	26.24 (7.85)	0.08		
	RCG	30.08 (7.14)	28.58 (7.95)	0.46	^a^ 0.80 ^b^ 0.61	^a^ 0.47 ^b^ 0.24
	RYG	29.00 (12.48)	27.08 (6.01)	0.62		
	HCG	26.67 (9.18)	23.42 (3.75)	0.10	^a^ 0.75	^a^ 0.67
	HYG	25.55 (7.46)	28.55 (4.66)	0.18	^b^ 0.01	^b^ 0.01
Calcium, mg/dl	CCG	8.85 (0.45)	9.07 (0.48)	0.24	^a^ 0.53 ^b^ 0.61	^a^ 0.87 ^b^ 0.58
	CYG	8.95 (0.27)	8.95 (0.63)	0.99		
	RCG	9.30 (0.80)	9.30 (0.49)	1.00	^a^ 0.22 ^b^ 0.75	^a^ 0.67 ^b^ 0.70
	RYG	8.93 (0.63)	9.24 (0.47)	0.13		
	HCG	8.72 (0.18)	8.74 (0.19)	0.75	^a^ 0.34 ^b^ 0.27	^a^ 0.55 ^b^ 0.47
	HYG	8.82 (0.28)	8.83 (0.20)	0.88		
Procalcitonin, ng/ml	CCG	0.05 (0.03)	0.05 (0.05)	0.95	^a^ 0.12 ^b^ 0.27	^a^ 0.34 ^b^ 0.98
	CYG	0.03 (0.01)	0.03 (0.02)	0.52		
	RCG	0.03 (0.02)	0.10 (0.22)	0.58	^a^ 0.10 ^b^ 0.43	^a^ 0.18 ^b^ 0.82
	RYG	0.20 (0.49)	0.19 (0.38)	0.97		
	HCG	0.12 (0.29)	0.04 (0.01)	0.65	^a^ 0.92 ^b^ 0.11	^a^ 0.11 ^b^ 0.72
	HYG	0.03 (0.01)	0.04 (0.04)	0.78		
Cortisol, ng/ml	CCG	281.00 (57.50)	258.67 (105.75)	0.65	^a^ 0.24	^a^ 0.47
	CYG	333.67 (85.28)	292.67 (77.21)	0.48	^b^ 0.54	^b^ 0.30
	RCG	306.60 (160.69)	248.50 (127.38)	0.38	^a^ 0.81	^a^ 0.80
	RYG	292.67 (82.11)	302.92 (141.59)	0.79	^b^ 0.40	^b^ 0.23
	HCG	170.12 (45.87)	215.10 (53.93)	0.10	^a^ 0.73 ^b^0.003	^a^ 0.84 ^b^ 0.004
	HYG	180.42 (87.06)	145.55 (39.86)	0.29		

Significance was observed at ≤ 0.05/18 = 0.003 after the Bonferroni correction for multiple comparison. P-value was adjusted with age and gender for between-group comparisons. CCG (N = 12), COVID-positive control group; CYG (N = 12), COVID-positive yoga group; RCG (N = 12), COVID-recovered control group; RYG (N = 12), COVID-recovered yoga group; HCG (N = 12), HCW control group; HYG (N = 11), HCW yoga group.

### Effect of intervention on neuropsychological parameters

Yogic breathing is known to improve psychological parameters such as stress, anxiety, awareness, and quality of life (18, 19). Nine neuropsychological questionnaires were used to evaluate these parameters. However, neuropsychological changes were not found significant in any of the groups after 15 days of the intervention (Supplementary Tables 3–5). The *Prakriti* type of participants infected with SARS-CoV-2 (COVID-positive and COVID-recovered) were predominantly *Kapha* (48.9 %), followed by *Pitta* (34.0 %), *Vata* (12.8 %), and a combination of *Kapha-Pitta* (4.3 %).

### Breathing technique improves exercise capacity

**Figure 2:**
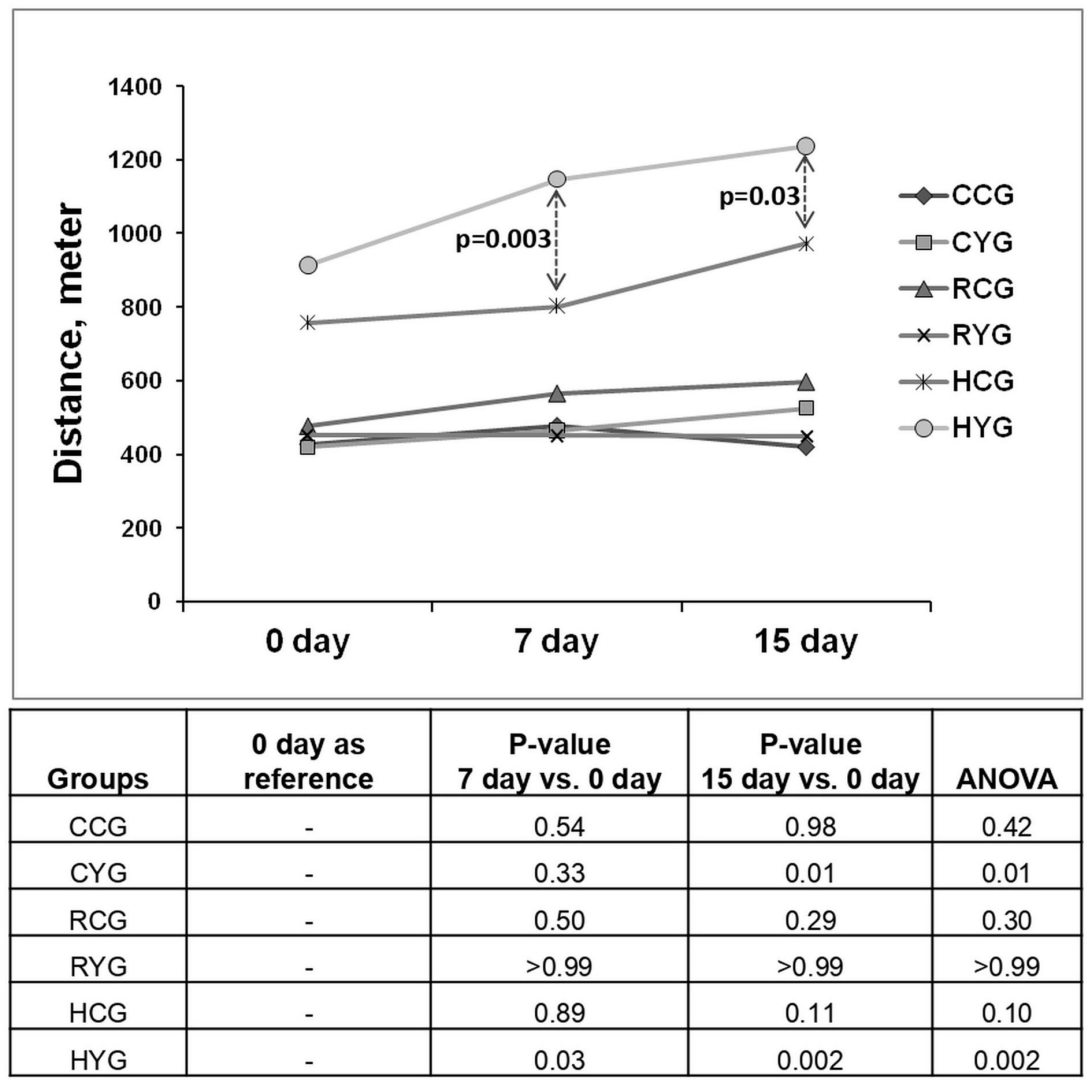
Distance covered in 6-min walk test. Significance was observed at *p* ≤ 0.05. *P*-values calculated for between-group comparisons were adjusted with age and gender. CCG (*N* = 10), COVID-positive control group; CYG (*N* = 12), COVID-positive yoga group; RCG (*N* = 8), COVID-recovered control group; RYG (*N* = 10), COVID-recovered yoga group; HCG (*N* = 7), HCW control group; HYG (*N* = 12), HCW yoga group.

The 6MWT showed increased exercise capacity and stamina among participants practicing SBT in the COVID-positive group and both SBT and LDBT in the HCW group. The distance covered was significantly more in the CYG and HYG on the 15th day as compared to the baseline (Figure 2). It was greater in the HYG on the 7th and 15th days as compared to HCG. Although a greater covered distance was observed in CYG than in CCG on the 15^th^ day, the difference was not significant (Figure 2). No specific trend was noticed in the clinical parameters measured before and after the 6MWT (Supplementary Table 6). Similarly, no comparable trend was observed in clinical parameters estimated during 1MSST (Supplementary Table 7).

### Effect of intervention on heart rate variability

The root mean square of the successive difference (RMSSD), which is a measure of HRV, was not significantly found to be altered in any group. Heart rate was noted to be lower (significant before the Bonferroni correction) in CYG than in CCG after intervention. Furthermore, high-frequency power (HF%) was significantly reduced in CYG. No changes were observed in the HRV of the COVID-recovered or HCW group (Supplementary Table 8).

Furthermore, there was no correlation of distance covered under 6MWT with neuropsychological and biochemical parameters.

The correlation between neuropsychological and biochemical parameters was also evaluated but no correlation was observed. Each intervention group was also analyzed based on *Prakriti* type. The investigated clinical, biochemical, and neuropsychological parameters were compared within each *Prakriti* type, and the three *Prakriti* types were compared with each other. However, no significant and conclusive observations were noted.

## Discussion

The breathing interventions in the COVID-positive patients, COVID-recovered patients, and HCWs show encouraging results with respect to a D-dimer and exercise capacity. WBC count was found to be increased in the COVID-positive subjects regardless of intervention because WBC count is low at the baseline in patients due to viral infection and it improves with health. We observed that D-dimer, a predictor of COVID-19 severity, was maintained below 0.50 μg/ml in CYG, but it was found to be increased in CCG. Elevated D-dimer with ≥0.50 μg/ml level indicates the formation and destruction of thrombus in the body (43–45). D-dimer was also decreased in RYG and HYG practicing breathing protocols (Table 1). Lowering D-dimer by yogic breathing may be helpful in reducing thrombosis and venous thromboembolism in patients with COVID-19 and in lowering the chances of vaccine-induced thrombotic thrombocytopenia in vaccinated individuals (46–49). These findings suggest that the severity of COVID-19 can possibly be modulated by practicing Yogic breathing techniques. However, a study on severe cases is warranted to validate these results in the future.

The 6MWT is a test that can aid in assessing the respiratory capacity, exercise, or functional capacity of patients with cardiopulmonary or pulmonary disease (50). Breathing intervention improved exercise capacity and stamina in CYG and HYG over a period of 15 days, which was noticed more in the yoga groups than in their respective control groups. Increased distance covered in CYG also suggests an improved prognosis (51, 52). In addition, HF% was found to be lowered in the CYG. Reduced HF% indicates decreased parasympathetic activity.

Integrating SBT and LBDT in COVID and post-COVID management appears to be beneficial and perhaps essential with emerging new variants every few months. The recent SARS-CoV-2 variant, omicron, which originated in November 2021, is resistant to the existing treatment and vaccination as it has been reported to not being neutralized by antibodies present in vaccinated and COVID-positive patients' sera (4, 6). A booster dose of Pfizer was able to generate neutralizing response against omicron but it was lower than that against the delta variant (6). Hence, exploring different strategies in COVID-19, including controlled breathing achieved by mindfulness meditation (SBT and LBDT), is useful. Immunity can be enhanced by meditation possibly by upregulating immune genes, which are dysfunctional in severe COVID-19, without affecting inflammatory genes (53).

In addition, we report that individuals with *Prakriti*-type *Kapha* are at a higher risk of getting infected with SARS-CoV-2, which was not reported earlier. The study suggests COVID-19 susceptibility of patients of *Kapha* dominant groups, followed by patients of *Pitta, Vata*, and a combination of *Kapha-Pitta*. In a recent study, Rajan et al. reported the highest frequency of *Vata-Kapha Prakriti* among 117 COVID-positive patients (54). Ayurgenomic approach has earlier shown that *Prakriti* type bears a relationship with genetic pathways. As reported in rheumatic arthritis, the *Pitta* group showed increased expression of oxidative stress genes and the *Vata* group was characterized by overexpression of inflammatory genes. This indicates the unexplored determinants of genetic pathways involved in disease (55). *Prakriti* types have been found to have unique genetic variability that could address phenotypic heterogeneity of patients and understand their disease susceptibility, conditioning, and predictive health outcomes (55, 56). Different trajectories of immune response, thrombosis, and bleeding are reported among the *Prakriti* types based on genetic variations in IFIT5 and SERPINA10 genes (57). These biological pathways are crucial in the case of COVID-19 and other infectious and noninfectious diseases. Moreover, genetic variants in HLA vary in different *Prakriti* types which is associated with susceptibility toward COVID-19 (33–35). *Prakriti* types also influence an individual's response toward an intervention, as it has been shown that only *Pitta Prakriti* individuals show an increment in oxygen saturation at high altitude after mindfulness meditation (58). We segregated the subjects according to *Prakriti* and evaluated neuropsychological and biochemical changes due to intervention in each type. However, significant trends could not be observed due to a small sample size. It would be attractive to investigate further whether types or combinations of complications, such as severe inflammation and requirement of ventilator, arise in various *Prakriti* types of patients with COVID-19. This may help examine the outcomes arising from dietary, environmental factors, yoga regimen, and lifestyle-related changes.

We highlight here some limitations that may affect the interpretation of the study. The sample size of the study was small due to a significant number of patients dropping out due to fear of growing mortality rate worldwide at that time. The fear that unconventional interventions (like SBT and LBDT) may have adverse effects on the prognosis, coupled with the scarce medical resources due to the COVID-19 outbreak, may have affected the outcomes. No significant neuropsychological changes were noticed in any of the groups. This may be due to the short duration of interventions. There was no difference between control and yoga groups in neuropsychology because the participants, irrespective of control or yoga groups, were known to the symptoms, vaccines, and underlying risks of COVID-19 through media and were under similar surroundings. Breathing exercise is generally more effective for symptomatic anxiety and when it is a trait in the individual, the breathing exercise did not have any effect on the situational anxiety created by COVID-19. One can assume that the large number of questionnaire may have disengaged the subjects from active participation. However, we tried to reduce disengagement by giving short intervals during questionnaire filling to avoid the fatigability effect. Doubts about the vaccine and the general uncertainty of the effectiveness of available medical help at that time may have influenced the psychological parameters and no change was observed. However, it has been reported that those who practice yoga are less stressed and could successfully cope with restrictions and adversity associated with COVID-19 during lockdown (13). Despite these limitations, we observed favorable outcomes in patients with COVID-19, a decrease in COVID-19 severity indicated by some of the biomarkers. The study could not include severe COVID-19 cases, as these patients were often mechanically supported for breathing and were unable to adapt to the breathing techniques. A shorter duration of intervention may have limited the scope of the study and might also have impacted the effectiveness of the interventions. A consistent practice of these breathing techniques over 4–6 weeks may have improved the outcomes, especially in measuring the neuropsychological outcomes.

In conclusion, the study revealed that breathing intervention lowered the D-dimer. Hence, the intervention was capable of reducing severity in mild to moderate cases of COVID-19. In addition, the exercise capacity and stamina of the patients improved, as a result of a breathing intervention protocol. The intervention can be easily administered to patients either in person or online. Therefore, this breathing protocol can be considered for integration in the management of COVID-positive and post-COVID cases.

## Data availability statement

The original contributions presented in the study are included in the article/Supplementary material, further inquiries can be directed to the corresponding author/s.

## Ethics statement

The studies involving human participants were reviewed and approved by Institutes Ethics Committee, PGIMER Chandigarh (IEC no. IEC-05/2020-1646). The patients/participants provided their written informed consent to participate in this study.

## Author contributions

MR collected, validated and analyzed the data and wrote the first draft with Figures and Tables. Literature search and data interpretation were done by MR and AA. AA, PA, and BS conceptualized the study. AA designed the study. AA and VS edited and critically reviewed the manuscript. AB, GP, PA, AA, VK, PMah, MS, NP, PMal, SGo, KK, NS, and SA facilitated the study and critically reviewed the manuscript. KM, PV, ND, SGu, PMe, and PN collected the data, performed tests, and validated the data. RK validated and analyzed the data. All the authors have read and agreed to the final manuscript and authorship order.

## Funding

Ministry of AYUSH, Government of India, New Delhi, India funded this study under grant no. Z-28015/48/2020-HPC (EMR)-AYUSH. The funds were provided for the execution of the study.

## Conflict of interest

The authors declare that the research was conducted in the absence of any commercial or financial relationships that could be construed as a potential conflict of interest.

## Publisher's note

All claims expressed in this article are solely those of the authors and do not necessarily represent those of their affiliated organizations, or those of the publisher, the editors and the reviewers. Any product that may be evaluated in this article, or claim that may be made by its manufacturer, is not guaranteed or endorsed by the publisher.
