# Supplementary material for: Effect of breathing intervention in patients with COVID and healthcare workers
Source: Front Public Health. 2022 Sep 30;10:945988. doi: 10.3389/fpubh.2022.945988 (PMC9561424; doi:10.3389/fpubh.2022.945988)
Supplement: Supplementary file 1 [file Data_Sheet_1.docx]

**Supplementary Table 1.Short Breathing Technique (SBT)**

| **Time Interval**: 3-5 minutes | | |
| --- | --- | --- |
| **Preparation**: eyes closed, sit with cross legs | | |
| **1** | Powerful inhalation and exhalation (*Bhastrika Pranayama*) 21 times | Constrictions from the throat with no abdominal jerks and eyes closed. |
| **2** | Sit relaxed with the fullness of breath as long as comfortably possible (at least for 30 secs). | Mouth and eyes closed |

**Supplementary Table 2. Socio-demographics of the study groups**

| **Socio-demographics** | **COVID positive control group (CCG), N=12** | **COVID positive yoga group (CYG), N=12** | **P-value** |
| --- | --- | --- | --- |
| Age | 35.75 (13.12); N=12 | 26.92 (9.18); N=12 | 0.99 |
| BMI | 24.78 (4.75); N=10 | 23.72 (3.90); N=12 | 0.79 |
| Gender | Female = 3  Male = 9 | Female = 6  Male = 6 | 0.21 |
| Smoking | Yes = 1  No = 11 | Yes = 2  No = 10 | 0.54 |
| Alcohol | Yes = 3  No = 9 | Yes = 4  No = 8 | 0.65 |
| Diet | Non-vegetarian = 11  Eggetarian = 0  Vegetarian = 1 | Non-vegetarian = 9  Eggetarian = 1  Vegetarian = 2 | 0.46 |
| Physical Activity | No=0  Minimum = 0  Moderate = 9  Very active =3 | No=0  Minimum = 1  Moderate = 8  Very active = 3 | 0.59 |
| Sleeping Hours | less than 5 hours = 2  5-6 hours = 3  7-8 hours = 5  more than 8 hours = 2 | less than 5 hours = 0  5-6 hours = 5  7-8 hours = 4  more than 8 hours = 3 | 0.42 |
| **Socio-demographics** | **COVID Recovered control group (RCG), N=12** | **COVID Recovered yoga group (RYG), N=12** | **P-value** |
| Age | 31.83 (11.22); N=12 | 39.58 (13.83); N=12 | **0.04** |
| BMI | 23.68 (2.70); N=12 | 25.37 (5.29); N=12 | 0.15 |
| Gender | Female = 7  Male = 5 | Female = 7  Male = 5 | 1.00 |
| Smoking | Yes = 2  No = 10 | Yes = 0  No = 12 | 0.07 |
| Alcohol | Yes = 2  No = 10 | Yes = 3  No = 9 | 0.62 |
| Diet | Non-Vegetarian = 6  Eggetarian = 3  Vegetarian = 3 | Non-Vegetarian = 8  Eggetarian = 3  Vegetarian = 1 | 0.53 |
| Physical Activity | No = 1  Minimum = 1  Moderate = 8  Very active = 2 | No = 0  Minimum = 4  Moderate = 5  Very active = 3 | 0.30 |
| Sleeping Hours | less than 5 hours = 0  5-6 hours = 5  7-8 hours = 5  more than 8 hours = 2 | less than 5 hours = 0  5-6 hours = 2  7-8 hours = 8  more than 8 hours = 1  Missing = 1 | 0.35 |
| **Socio-demographics** | **HCW control group (HCG), N=12** | **HCW yoga group (HYG), N=12** | **P-value** |
| Age | 34.17 (7.49); N=12 | 35.08 (9.52); N=12 | 0.37 |
| BMI | 27.31 (5.56); N=10 | 24.51 (4.85); N=10 | 0.95 |
| Gender | Female = 8  Male = 4 | Female = 5  Male = 7 | 0.22 |
| Smoking | Yes =1  No = 11 | Yes = 0  No = 12 | 0.21 |
| Alcohol | Yes = 1  No = 10  Missing = 1 | Yes = 4  No = 8 | 0.17 |
| Diet | Non-Vegetarian = 4  Eggetarian = 2  Vegetarian = 3  Missing = 3 | Non-Vegetarian = 6  Eggetarian = 0  Vegetarian = 6 | **0.03** |
| Physical Activity | No = 1  Minimum = 2  Moderate = 2  Very active = 2  Missing = 5 | No = 0  Minimum = 3  Moderate = 6  Very active = 2  Missing = 1 | 0.17 |
| Sleeping Hours | less than 5 hours = 0  5-6 hours = 2  7-8 hours = 5  more than 8 hours = 3  Missing = 2 | less than 5 hours = 0  5-6 hours = 4  7-8 hours = 7  more than 8 hours = 0  Missing = 1 | 0.23 |
| Age and BMI are represented as mean (standard deviation). Number of subjects (N) is mentioned for the each socio-demographic parameters. | | | |

**Supplementary Table 3. Neuropsychological parameters in COVID positive study subjects**

| **Neuropsychological Parameters** | | **COVID positive Yoga Group (CYG). N=12** | | | **COVID positive Control Group (CCG), N=8** | | | **CYG VS CCG**  **^a^ At baseline**  **^b^ On 15th day** | |
| --- | --- | --- | --- | --- | --- | --- | --- | --- | --- |
|  |  | **Baseline** | **15^th^ Day** | **P-value** | **Baseline** | **15^th^ Day** | **P-value** | **P-value** | **Adjusted P-value** |
| **PSS** | | 17.25 (7.26) | 15.75 (7.10) | 0.29 | 18.88 (4.76) | 18.38 (3.66) | 0.75 | **^a^**0.59  **^b^** 0.35 | **^a^** 0.72  **^b^** 0.41 |
| **POMS** | **Tension** | 4.25 (5.29) | 3.00 (2.95) | 0.17 | 5.57 (5.00) | 4.71 (5.47) | 0.60 | **^a^**0.60  **^b^** 0.38 | **^a^** 0.51  **^b^** 0.55 |
|  | **Depression** | 3.16 (5.31) | 3.25 (5.28) | 0.93 | 7.73 (8.68) | 3.43 (3.95) | 0.09 | **^a^**0.20  **^b^** 0.94 | **^a^** 0.69  **^b^** 0.75 |
|  | **Anger** | 1.00 (2.00) | 3.33 (4.94) | 0.05 | 3.29 (3.30) | 3.29 (5.77) | 1.00 | **^a^**0.08  **^b^** 0.98 | **^a^** 0.46  **^b^** 0.92 |
|  | **Fatigue** | 3.33 (3.94) | 3.16 (4.28) | 0.82 | 5.71 (3.45) | 3.71 (3.95) | 0.08 | **^a^** 0.20  **^b^** 0.79 | **^a^** 0.53  **^b^** 0.63 |
|  | **Confusion** | 3.50 (2.91) | 4.25 (4.29) | 0.43 | 4.00 (1.04) | 3.71 (4.11) | 0.65 | **^a^**0.73  **^b^** 0.79 | **^a^** 0.55  **^b^** 0.95 |
|  | **Vigour** | 14.83 (7.45) | 22.00 (6.65) | 0.01 | 10.14 (6.62) | 16.43 (7.04) | 0.004 | **^a^**0.19  **^b^** 0.10 | **^a^** 0.07  **^b^** 0.02 |
| **MAAS** | | 4.31 (0.81) | 4.08 (1.11) | 0.20 | 3.84 (1.86) | 4.37 (1.47) | 0.39 | **^a^**0.45  **^b^** 0.63 | **^a^** 0.05  **^b^** 0.92 |
| **JSDPES** | | 5.16 (1.04) | 4.83 (1.37) | 0.26 | 4.35 (1.51) | 4.75 (1.42) | 0.47 | **^a^**0.17  **^b^** 0.90 | **^a^** 0.03  **^b^** 0.33 |
| **PHQ4** | | 3.25 (2.73) | 2.00 (2.37) | 0.11 | 4.38 (3.02) | 3.50 (1.77) | 0.54 | **^a^**0.40  **^b^** 0.15 | **^a^** 0.70  **^b^** 0.19 |
| **WEMWBS** | | 50.90 (9.32) | 52.63 (8.43) | 0.23 | 45.50 (10.16) | 54.13 (10.09) | 0.05 | **^a^**0.25  **^b^** 0.73 | **^a^** 0.04  **^b^** 0.30 |
| **BRS** | | 3.34 (0.47) | 3.22 (0.45) | 0.39 | 3.40 (0.42) | 3.48 (0.34) | 0.53 | **^a^** 0.79  **^b^** 0.22 | **^a^** 0.64  **^b^** 0.90 |
| **PTGI** | | 55.00 (27.72) | 50.28 (25.93) | 0.56 | 55.50 (31.20) | 58.00 (26.31) | 0.74 | **^a^**0.58  **^b^** 0.58 | **^a^** 0.75  **^b^** 0.92 |
| **WHOQOL** | | 83.45 (6.42) | 85.71 (10.58) | 0.28 | 85.60 (7.57) | 91.38 (8.57) | 0.08 | **^a^** 0.51  **^b^** 0.22 | **^a^** 0.74  **^b^** 0.98 |
| **Domain 1**  **(physical health)** | | 21.75 (2.05) | 23.58 (3.99) | 0.06 | 22.63 (2.67) | 24.88 (3.44) | 0.08 | **^a^** 0.42  **^b^** 0.46 | **^a^** 0.79  **^b^** 0.47 |
| **Domain 2 (psychological health)** | | 21.00 (1.65) | 21.83 (3.01) | 0.18 | 20.98 (2.24) | 23.13 (2.64) | 0.13 | **^a^** 0.98  **^b^** 0.34 | **^a^** 0.94  **^b^** 0.46 |
| **Domain 3 (social relationships)** | | 11.96 (2.30) | 10.54 (3.19) | 0.05 | 12.38 (1.41) | 12.00 (0.93) | 0.44 | **^a^** 0.65  **^b^** 0.16 | **^a^** 0.63  **^b^** 0.44 |
| **Domain 4 (environmental health)** | | 28.75 (3.19) | 29.75 (2.96) | 0.20 | 29.63 (3.34) | 31.38 (4.24) | 0.11 | **^a^** 0.57  **^b^** 0.32 | **^a^** 0.56  **^b^** 0.56 |

Data is represented in mean (standard deviation). Significance was observed at p≤0.05/18 = 0.003 after Bonferroni's correction for multiple comparison. P-value was adjusted with age and gender for between-group comparisons.

**Supplementary Table 4. Neuropsychological parameters in COVID recovery study subjects**

| **Neuropsychological Parameters** | | **COVID recovery Yoga Group (RYG), N=12** | | | **COVID recovery Control Group (RCG), N=12** | | | **RYG VS RCG**  **^a^ At baseline**  **^b^ On 15th day** | |
| --- | --- | --- | --- | --- | --- | --- | --- | --- | --- |
|  |  | **Baseline** | **15^th^ Day** | **P-value** | **Baseline** | **15^th^ Day** | **P-value** | **P-value** | **Adjusted P-value** |
| **PSS** | | 14.67 (7.08) | 16.17 (6.64) | 0.18 | 19.18 (8.26) | 16.55 (4.93) | 0.13 | ^a^0.17  ^b^0.88 | ^a^ 0.15  ^b^ 0.98 |
| **POMS** | **Tension** | 2.64 (2.80) | 2.91 (3.39) | 0.79 | 7.18 (4.33) | 6.82 (4.12) | 0.74 | ^a^0.01  ^b^ 0.02 | ^a^ 0.004  ^b^ 0.03 |
|  | **Depression** | 2.09 (3.62) | 5.00 (6.39) | 0.19 | 8.27 (6.90) | 6.91 (6.80) | 0.20 | ^a^ 0.02  ^b^ 0.50 | ^a^ 0.004  ^b^ 0.43 |
|  | **Anger** | 2.45 (3.24) | 2.73 (4.36) | 0.84 | 6.09 (4.18) | 5.55 (5.41) | 0.66 | ^a^ 0.03  ^b^ 0.19 | ^a^ 0.01  ^b^ 0.12 |
|  | **Fatigue** | 3.27 (3.72) | 2.64 (3.04) | 0.58 | 6.55 (5.05) | 4.82 (3.54) | 0.21 | ^a^ 0.10  ^b^ 0.14 | ^a^ 0.14  ^b^ 0.17 |
|  | **Confusion** | 2.82 (2.36) | 3.45 (3.64) | 0.64 | 7.45 (3.47) | 6.64 (4.43) | 0.57 | ^a^ **0.002**  ^b^ 0.08 | ^a^ 0.01  ^b^ 0.19 |
|  | **Vigour** | 21.09 (7.37) | 17.00 (8.05) | 0.26 | 17.64 (6.04) | 18.55 (5.99) | 0.51 | ^a^ 0.24  ^b^ 0.62 | ^a^ 0.30  ^b^ 0.36 |
| **MAAS** | | 4.63 (0.86) | 4.25 (1.10) | 0.05 | 3.94 (1.14) | 3.98 (0.89) | 0.88 | ^a^ 0.12  ^b^ 0.54 | ^a^ 0.22  ^b^ 0.98 |
| **JSDPES** | | 4.97 (0.71) | 4.76 (0.75) | 0.29 | 4.57 (1.36) | 4.74 (1.02) | 0.63 | ^a^ 0.38  ^b^ 0.95 | ^a^ 0.17  ^b^ 0.95 |
| **PHQ4** | | 2.17 (2.25) | 2.08 (2.11) | 0.85 | 3.50 (2.84) | 2.92 (1.73) | 0.42 | ^a^ 0.22  ^b^ 0.30 | ^a^ 0.22  ^b^ 0.40 |
| **WEMWBS** | | 53.00 (8.83) | 50.42 (8.18) | 0.25 | 46.42 (9.75) | 52.17 (6.56) | 0.07 | ^a^ 0.10  ^b^ 0.57 | ^a^ 0.14  ^b^ 0.23 |
| **BRS** | | 3.36 (0.58) | 3.40 (0.46) | 0.71 | 3.15 (0.74) | 3.21 (0.69) | 0.69 | ^a^ 0.45  ^b^ 0.43 | ^a^ 0.35  ^b^ 0.20 |
| **PTGI** | | 68.71 (20.51) | 69.43 (19.77) | 0.83 | 52.86 (30.63) | 52.71 (18.25) | 0.99 | ^a^ 0.28  ^b^ 0.13 | ^a^ 0.27  ^b^ 0.57 |
| **WHOQOL** | | 86.58 (9.20) | 86.50 (11.51) | 0.97 | 84.70 (10.56) | 83.60 (9.96) | 0.67 | ^a^ 0.66  ^b^ 0.54 | ^a^ 0.41  ^b^ 0.71 |
| **Domain 1**  **(physical health)** | | 22.58 (3.45) | 23.25 (3.79) | 0.53 | 23.50 (2.37) | 23.20 (2.62) | 0.70 | ^a^ 0.49  ^b^ 0.97 | ^a^ 0.50  ^b^ 0.76 |
| **Domain 2 (psychological health)** | | 21.50 (2.07) | 21.08 (2.50) | 0.41 | 20.30 (2.91) | 20.20 (2.74) | 0.88 | ^a^ 0.27  ^b^ 0.44 | ^a^ 0.15  ^b^ 0.61 |
| **Domain 3 (social relationships)** | | 11.42 (2.57) | 10.92 (1.93) | 0.29 | 9.50 (2.17) | 9.90 (3.03) | 0.51 | ^a^ 0.08  ^b^ 0.35 | ^a^ 0.09  ^b^ 0.51 |
| **Domain 4 (environmental health)** | | 31.08 (3.78) | 31.25 (4.86) | 0.84 | 31.40 (4.67) | 30.30 (4.74) | 0.37 | ^a^ 0.86  ^b^ 0.65 | ^a^ 0.77  ^b^ 0.68 |

Data is represented in mean (standard deviation). Significance was observed at p≤0.05/18=0.003 after Bonferroni's correction for multiple comparisons. P-value was adjusted with age and gender for between-group comparisons.

**Supplementary Table 5. Neuropsychological parameters in HCW study subjects**

| **Neuropsychological Parameters** | | **HCW Yoga Group (HYG), N=9** | | | **HCW Control Group (HCG), N=12** | | | **HYG**  **VS HCG**  **^a^At baseline**  **^b^ On 15th day** | |
| --- | --- | --- | --- | --- | --- | --- | --- | --- | --- |
|  |  | **Baseline** | **15^th^ Day** | **P-value** | **Baseline** | **15^th^ Day** | **P-value** | **P-value** | **Adjusted P-value** |
| **PSS** | | 17.88 (2.36) | 20.00 (4.31) | 0.25 | 20.64 (3.50) | 17.64 (4.46) | 0.19 | ^a^ 0.07  ^b^0.26 | ^a^ 0.07  ^b^ 0.21 |
| **POMS** | **Tension** | 4.67 (3.72) | 3.83 (3.06) | 0.79 | 5.13 (3.83) | 4.88 (4.85) | 0.85 | ^a^ 0.83  ^b^0.65 | ^a^ 0.53  ^b^ 0.63 |
|  | **Depression** | 3.83 (3.97) | 2.50 (2.26) | 0.19 | 5.13 (5.82) | 7.00 (6.41) | 0.51 | ^a^ 0.65  ^b^0.10 | ^a^ 0.38  ^b^ 0.08 |
|  | **Anger** | 4.00 (3.58) | 3.83 (2.04) | 0.84 | 5.13 (3.27) | 5.75 (4.33) | 0.65 | ^a^ 0.55  ^b^0.30 | ^a^ 0.11  ^b^ 0.15 |
|  | **Fatigue** | 5.67 (4.84) | 3.33 (1.86) | 0.58 | 4.75 (4.65) | 5.63 (3.81) | 0.58 | ^a^ 0.73  ^b^0.20 | ^a^ 0.76  ^b^ 0.11 |
|  | **Confusion** | 4.33 (2.58) | 4.83 (3.06) | 0.64 | 3.00 (1.85) | 4.13 (3.72) | 0.45 | ^a^ 0.28  ^b^0.71 | ^a^ 0.64  ^b^ 0.62 |
|  | **Vigour** | 20.50 (6.50) | 14.50 (8.62) | 0.26 | 16.75 (7.61) | 11.00 (6.61) | 0.15 | ^a^ 0.35  ^b^0.40 | ^a^ 0.99  ^b^ 0.74 |
| **MAAS** | | 3.83 (0.63) | 3.87 (0.72) | 0.87 | 4.26 (0.68) | 3.59 (1.12) | 0.18 | ^a^ 0.17  ^b^0.55 | ^a^ 0.27  ^b^ 0.57 |
| **JSDPES** | | 5.17 (0.57) | 5.28 (0.55) | 0.71 | 5.00 (1.28) | 4.74 (1.44) | 0.59 | ^a^ 0.72  ^b^0.28 | ^a^ 0.43  ^b^ 0.22 |
| **PHQ4** | | 4.00 (3.84) | 2.89 (2.20) | 0.41 | 4.80 (3.77) | 3.40 (2.55) | 0.18 | ^a^ 0.65  ^b^0.65 | ^a^ 0.67  ^b^ 0.42 |
| **WEMWBS** | | 54.50 (5.83) | 50.00 (8.37) | 0.19 | 51.63 (10.86) | 55.88 (9.91) | 0.44 | ^a^ 0.52  ^b^0.22 | ^a^ 0.62  ^b^ 0.29 |
| **BRS** | | 2.81 (0.37) | 3.37 (0.67) | 0.08 | 3.02 (0.74) | 3.26 (1.09) | 0.66 | ^a^ 0.47  ^b^0.81 | ^a^ 0.49  ^b^ 0.85 |
| **PTGI** | | 64.40 (8.62) | 53.20 (25.07) | 0.46 | 67.40 (18.45) | 76.40 (16.83) | 0.24 | ^a^ 0.75  ^b^0.12 | ^a^ 0.59  ^b^ 0.26 |
| **WHOQOL** | | 80.75 (11.21) | 85.88 (10.55) | 0.36 | 85.83 (9.82) | 83.67 (12.67) | 0.70 | ^a^ 0.30  ^b^0.69 | ^a^ 0.38  ^b^ 0.10 |
| **Domain 1**  **(physical health)** | | 21.88 (3.18) | 22.13 (3.04) | 0.84 | 24.17 (2.17) | 24.25 (3.22) | 0.94 | ^a^ 0.07  ^b^0.16 | ^a^ 0.20  ^b^ 0.84 |
| **Domain 2 (psychological health)** | | 19.88 (3.80) | 21.50 (1.51) | 0.11 | 21.58 (3.92) | 21.00 (3.10) | 0.71 | ^a^ 0.35  ^b^0.64 | ^a^ 0.89  ^b^ 0.84 |
| **Domain 3 (social relationships)** | | 11.25 (3.24) | 11.25 (3.28) | 1.00 | 11.17 (3.01) | 11.00 (2.76) | 0.88 | ^a^ 0.95  ^b^0.86 | ^a^ 0.08  ^b^ 0.62 |
| **Domain 4 (environmental health)** | | 27.75 (5.37) | 31.00 (4.50) | 0.29 | 28.92 (3.80) | 27.42 (6.46) | 0.52 | ^a^ 0.58  ^b^0.19 | ^a^ 0.80  ^b^ 0.32 |

Data is represented in mean (standard deviation). Significance was observed at ≤0.05/18 = 0.003 after Bonferroni's correction for multiple comparison. P-value was adjusted with age and gender for between- group comparisons.

**Supplementary Table 6. Change in clinical parameters after 6-minute walk test**

| **Clinical parameters** | **Study groups** | **Difference in pre and post values of clinical parameters at three timepoints** | | | **Repeated Measure** | |
| --- | --- | --- | --- | --- | --- | --- |
|  |  | **Baseline** | **7th day** | **15th day** | **F** | **P-value** |
| **SBP** | **CCG** | 6.10 (9.18) | 0.60 (13.43) | -0.30 (8.04) | 0.004 | 0.95 |
|  | **CYG** | 3.25 (7.84) | 0.83 (9.93) | -0.25 (11.14) |  |  |
| **DBP** | **CCG** | 3.10 (3.31) | 0.90 (8.88) | 0.80 (5.16) | 0.65 | 0.43 |
|  | **CYG** | 3.92 (5.95) | 1.17 (6.21) | 3.75 (6.03) |  |  |
| **MAP** | **CCG** | 4.10 (3.98) | 0.80 (7.33) | 0.37 (2.83) | 0.23 | 0.64 |
|  | **CYG** | 3.69 (5.69) | 1.06 (6.94) | 2.42 (7.10) |  |  |
| **Pulse rate** | **CCG** | 9.20 (10.09) | 5.50 (18.11) | 10.70 (13.28) | 0.12 | 0.73 |
|  | **CYG** | 6.08 (10.34) | 4.33 (24.56) | 8.08 (11.71) |  |  |
| **SpO2** | **CCG** | -0.20 (2.82) | -2.00 (4.45) | -1.10 (3.81) | 0.02 | 0.88 |
|  | **CYG** | -0.83 (3.64) | -2.92 (6.27) | -0.83 (1.85) |  |  |
| **SBP** | **RCG** | 5.63 (8.70) | 3.88 (13.30) | -16.25 (24.94) | 6.69 | 0.02 |
|  | **RYG** | -1.60 (10.30) | 3.90 (8.01) | -2.40 (6.38) |  |  |
| **DBP** | **RCG** | -1.00 (5.01) | -0.75 (10.38) | -1.88 (5.11) | 3.05 | 0.10 |
|  | **RYG** | -2.60 (11.14) | 5.50 (7.35) | 0.90 (7.11) |  |  |
| **MAP** | **RCG** | 1.21 (2.55) | 0.79 (10.04) | -6.67 (10.31) | 5.41 | 0.03 |
|  | **RYG** | -2.27 (8.32) | 4.97 (6.22) | -0.20 (5.88) |  |  |
| **Pulse rate** | **RCG** | 27.75 (15.75) | 19.38 (26.73) | 5.38 (17.48) | 2.34 | 0.15 |
|  | **RYG** | 5.90 (13.97) | 12.80 (10.30) | 12.60 (21.13) |  |  |
| **SpO2** | **RCG** | -1.00 (1.93) | -5.50 (11.06) | -2.13 (3.72) | 2.48 | 0.14 |
|  | **RYG** | 0.20 (1.75) | -0.50 (2.27) | -0.90 (1.91) |  |  |
| **SBP** | **HCG** | 4.29 (10.34) | 0.57 (9.47) | -4.86 (20.94) | 7.93 | **0.01** |
|  | **HYG** | 4.33 (8.52) | 11.25 (11.20) | 7.08 (7.82) |  |  |
| **DBP** | **HCG** | 2.71 (9.95) | 1.86 (4.91) | -0.71 (12.61) | 2.49 | 0.13 |
|  | **HYG** | 5.67 (9.01) | 9.08 (8.54) | 4.67 (9.32) |  |  |
| **MAP** | **HCG** | 3.24 (9.10) | 1.43 (6.07) | -2.10 (13.28) | 5.89 | 0.03 |
|  | **HYG** | 5.22 (7.67) | 9.81 (7.96) | 5.47 (8.18) |  |  |
| **Pulse rate** | **HCG** | 18.86 (12.46) | 14.57 (17.28) | 13.57 (11.30) | <0.001 | 0.98 |
|  | **HYG** | 18.25 (14.79) | 14.92 (21.71) | 12.92 (11.45) |  |  |
| **SpO2** | **HCG** | 0.43 (1.51) | 0.43 (2.37) | 0.86 (2.54) | 0.75 | 0.40 |
|  | **HYG** | -1.00 (4.20) | -0.42 (2.43) | 0.25 (3.41) |  |  |

Data is represented in mean (standard deviation). Significance was observed at ≤0.05/5 = 0.01 after Bonferroni’s correction for multiple comparison.

**Supplementary Table 7. Change in clinical parameters after 1-minute sit-stand test**

| **Clinical parameters** | **Study groups** | **Difference in pre and post values of clinical parameters at three timepoints** | | | **Repeated Measure** | |
| --- | --- | --- | --- | --- | --- | --- |
|  |  | **Baseline** | **7th day** | **15th day** | **F** | **P-value** |
| **SBP** | **CCG** | 9.70 (12.35) | 5.80 (7.71) | 8.30 (11.13) | 0.31 | 0.58 |
|  | **CYG** | 8.25 (15.93) | 10.00 (10.71) | 7.58 (9.57) |  |  |
| **DBP** | **CCG** | 4.00 (7.21) | -1.10 (3.51) | -3.40 (5.17) | 4.30 | 0.05 |
|  | **CYG** | 2.75 (7.50) | 4.17 (8.19) | 1.33 (9.79) |  |  |
| **MAP** | **CCG** | 5.90 (7.73) | 1.20 (3.16) | 0.50 (6.25) | 2.85 | 0.11 |
|  | **CYG** | 4.58 (8.15) | 6.11 (6.90) | 3.42 (8.42) |  |  |
| **Pulse rate** | **CCG** | 14.50 (27.69) | 23.20 (11.35) | 27.20 (11.70) | 0.002 | 0.97 |
|  | **CYG** | 14.08 (27.25) | 23.25 (23.50) | 27.58 (11.74) |  |  |
| **SpO2** | **CCG** | 0.20 (5.96) | -0.50 (3.98) | -1.30 (2.79) | 0.41 | 0.53 |
|  | **CYG** | -0.92 (2.27) | -1.17 (3.54) | 0.50 (3.68) |  |  |
| **SBP** | **RCG** | 7.50 (7.03) | 6.13 (7.00) | 6.38 (6.93) | 0.26 | 0.62 |
|  | **RYG** | 13.11 (8.43) | 8.67 (11.52 | 7.33 (12.29) |  |  |
| **DBP** | **RCG** | 1.50 (7.56) | -4.00 (10.52) | -0.63 (7.91) | 0.21 | 0.65 |
|  | **RYG** | -2.44 (3.57) | 0.44 (6.89) | -6.33 (9.81) |  |  |
| **MAP** | **RCG** | 3.50 (4.45) | -0.63 (6.71) | 1.71 (6.11) | 0.02 | 0.88 |
|  | **RYG** | 2.74 (3.29) | 3.19 (6.40) | -1.78 (8.26) |  |  |
| **Pulse rate** | **RCG** | 22.75 (12.98) | 13.38 (19.52) | 17.88 (6.96) | 1.11 | 0.31 |
|  | **RYG** | 20.11 (9.03) | 16.67 (18.08) | 22.22 (16.64) |  |  |
| **SpO2** | **RCG** | -0.25 (1.49) | -1.88 (3.56) | -0.13 (2.64) | 0.66 | 0.43 |
|  | **RYG** | -0.33 (2.45) | 1.00 (5.61) | -0.44 (2.01) |  |  |
| **SBP** | **HCG** | 15.57 (10.94) | 2.57 (5.77) | 11.57 (11.86) | 1.65 | 0.22 |
|  | **HYG** | 11.25 (9.93) | 11.42 (12.12) | 13.50 (12.30) |  |  |
| **DBP** | **HCG** | 0.71 (14.92) | 3.29 (10.52) | 0.71 (4.61) | 0.01 | 0.91 |
|  | **HYG** | -4.50 (9.36) | 0.42 (7.93) | 3.25 (10.42) |  |  |
| **MAP** | **HCG** | 5.67 (11.56) | 3.05 (7.48) | 4.33 (5.08) | 0.68 | 0.42 |
|  | **HYG** | 0.75 (7.93) | 4.08 (7.30) | 6.67 (8.32) |  |  |
| **Pulse rate** | **HCG** | 18.71 (12.47) | 16.43 (15.76) | 17.29 (15.92) | 0.68 | 0.42 |
|  | **HYG** | 19.92 (21.91) | 17.58 (12.35) | 25.58 (17.42) |  |  |
| **SpO2** | **HCG** | 0.57 (2.82) | -2.29 (6.34) | -0.29 (1.11) | 1.61 | 0.22 |
|  | **HYG** | -1.00 (1.60) | 0.33 (2.19) | 0.42 (2.02) |  |  |

Data is represented in mean (standard deviation). Significance was observed at ≤0.05/5 = 0.01 after Bonferroni’s correction for multiple comparison.

**Supplementary Table 8. Heart rate variability in the three study groups.**

| **Heart rate variability** | **COVID positive Yoga Group (CYG), N=8** | | | **COVID positive Group (CCG), N=11** | | | **CYG VS CCG**  **^a^At Baseline**  **^b^on 15^th^ day** | |
| --- | --- | --- | --- | --- | --- | --- | --- | --- |
|  | **Baseline** | **15^th^ Day** | **P-value** | **Baseline** | **15^th^ Day** | **P-value** | **P-value** | **Adjusted P-value** |
| R-R interval, ms | 1278.25 (1615.63) | 1756.88 (1466.20) | 0.60 | 902.00 (568.41) | 718.64 (207.89) | 0.32 | ^a^0.48  ^b^ 0.09 | ^a^ 0.25  ^b^ 0.09 |
| Root Mean Square of the Successive Differences (RMSSD), ms | 673.81 (1618.68) | 1060.09 (1615.13) | 0.69 | 525.63 (1002.24) | 61.39 (64.26) | 0.25 | ^a^0.93  ^b^0.12 | ^a^0.57  ^b^0.13 |
| Heart rate, bpm | 83.38 (45.46) | 57.13 (34.19) | 0.28 | 82.73 (29.81) | 89.00 (21.98) | 0.51 | ^a^ 0.97  ^b^0.04 | ^a^ 0.74  ^b^ 0.07 |
| High Frequency power (HF), % | 56.86 (16.53) | 28.91 (20.04) | **0.01** | 38.21 (22.06) | 31.90 (25.20) | 0.50 | ^a^ 0.06  ^b^ 0.78 | ^a^ 0.09  ^b^ 0.63 |
| Low Frequency power (LF), % | 39.42 (14.73) | 48.10 (20.65) | 0.42 | 49.03 (15.22) | 29.87 (23.25) | 0.08 | ^a^ 0.19  ^b^ 0.09 | ^a^ 0.16  ^b^ 0.16 |
| **Heart rate variability** | **COVID recovered Yoga Group (RYG), N=8** | | | **COVID recovered Control Group (RCG), N=8** | | | **RYG VS RCG**  **^a^At Baseline**  **^b^on 15^th^ day** | |
|  | **Baseline** | **15^th^ Day** | **P-value** | **Baseline** | **15^th^ Day** | **P-value** | **P-value** | **Adjusted P-value** |
| R-R interval, ms | 689.63 (77.58) | 723.00 (103.32) | 0.06 | 740.38 (108.73) | 788.75 (113.28) | 0.35 | ^a^ 0.30  ^b^ 0.24 | ^a^ 0.20  ^b^ 0.23 |
| Root Mean Square of the Successive Differences (RMSSD), ms | 18.74 (6.78) | 23.52 (9.22) | 0.02 | 28.46 (14.59) | 39.44 (22.88) | 0.21 | ^a^0.11  ^b^0.10 | ^a^0.60  ^b^0.48 |
| Heart rate, bpm | 87.88 (8.74) | 84.13 (10.51) | 0.07 | 82.50 (12.54) | 77.50 (11.64) | 0.34 | ^a^ 0.34  ^b^ 0.25 | ^a^ 0.20  ^b^ 0.23 |
| High Frequency power (HF), % | 24.30 (10.52) | 24.16 (12.70) | 0.93 | 25.40 (10.62) | 25.56 (6.20) | 0.97 | ^a^ 0.84  ^b^ 0.78 | ^a^ 0.75  ^b^ 0.67 |
| Low Frequency power (LF), % | 25.11 (11.87) | 21.60 (7.64) | 0.21 | 28.66 (13.32) | 30.61 (9.93) | 0.71 | ^a^ 0.58  ^b^ 0.06 | a 0.89  ^b^ 0.19 |
| **Heart rate variability** | **HCW Yoga Group (HYG), N=11** | | | **HCW Control Group (HCG), N=11** | | | **HYG VS HCG**  **^a^At Baseline**  **^b^on 15^th^ day** | |
|  | **Baseline** | **15^th^ Day** | **P-value** | **Baseline** | **15^th^ Day** | **P-value** | **P-value** | **Adjusted P-value** |
| R-R interval, ms | 710.45 (58.58) | 696.36 (47.43) | 0.30 | 721.55 (111.72) | 662.45 (83.85) | 0.08 | ^a^ 0.77  ^b^ 0.26 | ^a^ 0.59  ^b^ 0.60 |
| **Heart rate variability** | **HCW Yoga Group (HYG), N=11** | | | **HCW Control Group (HCG), N=11** | | | **HYG VS HCG**  **^a^At Baseline**  **^b^on 15^th^ day** | |
|  | **Baseline** | **15^th^ Day** | **P-value** | **Baseline** | **15^th^ Day** | **P-value** | **P-value** | **Adjusted P-value** |
| Root Mean Square of the Successive Differences (RMSSD), ms | 26.22 (12.20) | 24.50 (9.57) | 0.60 | 24.41 (22.10) | 23.22 (15.56) | 0.82 | ^a^0.82  ^b^0.33 | ^a^0.92  ^b^0.94 |
| Heart rate, bpm | 85.00 (7.60) | 86.55 (6.33) | 0.66 | 85.18 (14.44) | 92.09 (12.51) | 0.15 | ^a^ 0.97  ^b^ 0.21 | ^a^ 0.84  ^b^ 0.52 |
| High Frequency power (HF), % | 23.14 (12.42) | 22.63 (8.79) | 0.83 | 19.75 (10.39) | 21.93 (14.73) | 0.74 | ^a^ 0.50  ^b^ 0.89 | ^a^ 0.18  ^b^ 0.70 |
| Low Frequency power (LF), % | 30.88 (12.57) | 29.10 (12.42) | 0.68 | 29.09 (14.96) | 22.23 (11.32) | 0.26 | ^a^ 0.76  ^b^ 0.19 | ^a^ 0.78  ^b^ 0.34 |

Data is represented in mean (standard deviation). Significance was observed at ≤0.05/5 = 0.01 after Bonferroni's correction for multiple comparison. P-value was adjusted with age and gender for between group comparisons.

**Video Legends**

**Supplementary Video 1: Short Breathing Technique (SBT)**

**Supplementary Video 2: Longer Duration Breathing Technique (LDBT)**
